# Risk of metastasis among rib abnormalities on bone scans in breast cancer patients

**DOI:** 10.1038/srep09587

**Published:** 2015-05-05

**Authors:** Qin Li, Zhiqiang Chen, Yansheng Zhao, Xiuqing Li, Hong Pan, Tiansong Xia, Lin Chen, Zhaoqiang Xu, Wenbin Zhou, Xiaoan Liu

**Affiliations:** 1 Department of Breast Surgery, The First Affiliated Hospital with Nanjing Medical University, 300 Guangzhou Road, 210029 Nanjing, China; 2 Department of Nuclear Medicine, The First Affiliated Hospital with Nanjing Medical University, 300 Guangzhou Road, 210029 Nanjing, China

## Abstract

Bone scan abnormalities, especially rib lesions, are often confusing for physicians due to a high number of false-positive lesions. This study investigated risk factors that are associated with bone metastasis in 613 breast cancer patients with bone scan abnormalities. Significantly increased rates of bone metastasis were observed in patients with multiple lesions, large tumor sizes, and lymph node involvement. In addition, patients with concurrent lesions of rib and other sites exhibited a significant higher rate of metastatic disease compared to those with other site lesions (*P* = 0.009). In the subset of 324 patients with rib abnormalities, the rate of metastasis was extremely low in patients with pure rib lesions (1.2%; 95% CI: 0.1%–4.1%). Concurrent lesions of rib and other sites were more likely to be rib metastasis compared to pure rib lesions (*P* < 0.001). Moreover, multiple rib lesions and lesions located on bilateral ribs were more likely to be rib metastasis (P < 0.001). Our data suggest that patients with pure rib abnormalities could be recommended for follow-up only. However, if concurrent lesions of rib and other sites were detected on bone scans, additional radiological examinations should be performed to patients.

Bone is known to be one of the most common sites of relapse for breast cancer patients^1^,^2^,^3^. Several imaging modalities, including plain radiography (XR), computed tomography (CT), magnetic resonance imaging (MRI), skeletal scintigraphy (SS), positron emission tomography (PET), and single photon emission computed tomography (SPECT), are available for oncologists to detect bone metastasis. Compared to other modalities, SS is characteristic by its high sensitivity, easy accessibility, and providing whole-body imaging of bone metabolism. Therefore, it is more frequently used than the other modalities^4^,^5^, and breast cancer patients can benefit from a routine baseline bone scan and a regular follow-up^6^. However, due to the high rate of false-positive findings, the abnormalities detected on bone scans by SS, especially single lesion on images, can be quite confusing for physicians^7^,^8^,^9^. Risk factors for bone metastasis in patients with bone scan abnormalities have not been fully determined. Previous studies have reported that multiple hot spots and those located on spine on SS images usually indicate metastasis^10^,^11^. Besides, high levels of tumor marker CA153 have been reported to be associated with bone metastasis in patients with equivocal bone scans^12^. However, the association between clinical variables and bone metastasis among breast cancer patients with abnormal bone scans has not been systematically investigated.

Importantly, rib represents a common site where abnormalities would be detected on bone scans of breast cancer patients^10^. Trauma in surgery and complications of radiotherapy can also cause hot spots, causing a high false-positive rate of rib abnormalities detected on bone scans. Thus the diagnosis of lesions on ribs is difficult, especially for single or double rib abnormalities. The rate of bone metastasis among patients with solitary rib abnormality on bone scans ranged from 3.3% to 41% in previous reports^9^,^11^,^13^,^14^,^15^,^16^,^17^. Such a wide range may be due to small sample sizes and different inclusion criteria of those studies. Identifying predictors of bone metastasis among breast patients with rib abnormality on bone scans will help the management of these patients; however, very few studies have been conducted in this regard. To the best of our knowledge, only one study by Chen et al. in 2003 revealed that among breast cancer patients with one or two rib hotspots on bone scans, those with ten or more lymph node involved and those with a concurrent bone lesion other than the rib are more likely to have bone metastasis^16^. One limitation of that study was that the diagnosis of metastasis was not confirmed by other radiological examinations.

In the present study, using a large breast cancer patient population with bone scan abnormalities, we first evaluated the associations between clinical variables and bone metastasis among patients with overall bone scan abnormalities. We then specifically analyzed clinical risk factors associated with bone metastasis among patients with rib abnormalities.

## Results

A total of 613 female breast cancer patients with bone scan abnormalities were included in this study, among whom 126 (20.6%) were diagnosed as positive for metastatic disease. The median age was 52 years (range, 26–88 year). The mean follow-up time was 29.5 months (range, 0–312 months). The detailed information of these 613 patients was shown in Table 1.

### Risk factors associated with bone metastasis in patients with bone scan abnormalities

In univariate analyses of these 613 patients (Table 1), bone scan abnormalities in patients with a tumor size > 5 cm (9/23) were more likely to be bone metastasis than those in patients with a tumor size ≤ 2 cm (28/257) or 2–5 cm (42/228) (*P* < 0.001). In addition, bone scan abnormalities in patients with lymph node involvement (63/286) were more likely to be bone metastasis than those in patients without lymph node involvement (34/267) (*P* = 0.004).

Of these 613 patients, 173 patients had pure rib lesions, 289 had lesions on other sites, and 151 had combined lesions of rib and other sites on their bone scans. The rates of bone metastasis in these three groups were significantly different (*P* < 0.001). Importantly, the rate of bone metastasis was the lowest in patients with pure rib lesions (1.2%; 95% CI: 0.1%–4.1%). Furthermore, the rates of bone metastasis were 7.5%, 16.4% and 47.4% respectively in patients with single, double and ≥3 lesions on their bone scans, and a significant difference was observed in these three groups (*P* < 0.001).

No significant differences were observed in the rates of bone metastasis among patients with different ages (*P* = 0.467), menopausal status (*P* = 0.511), hormone receptors status (*P* = 0.517), Her2 status (*P* = 0.723) and molecular subtypes (*P* = 0.691).

In multivariate analyses (Table 1), the abnormalities on bone scans in patients with lymph node involvement were more likely to be metastatic lesions than those in patients without lymph node involvement (OR = 1.87; 95% CI = 1.04–3.34; *P* = 0.035). The abnormalities on bone scans in patients with a tumor size > 5 cm were more likely to be metastatic lesions than those in patients with a tumor size < 2 cm (OR = 3.44; 95% CI = 1.10–10.83; *P* = 0.034). Multiple lesions (≥3) were more likely to be metastatic lesions than solitary lesion (OR = 3.08; 95% CI = 1.32–7.21; *P* = 0.009). Compared to other site lesions, combined lesions of rib and other sites had a significant higher rate of metastatic disease (OR = 2.65; 95% CI = 1.28–5.51; *P* = 0.009).

### Characteristics of bone metastasis in 324 patients with rib lesions on bone scans

There were 324 patients with rib lesions on their bone scans. Of these patients, 79 (24.4%) were confirmed to be bone metastasis by CT or MRI. The detailed information about these 79 patients was shown in Supplement Table 1. Two patients with pure rib abnormalities were confirmed to have bone metastasis. One patient with concurrent abnormalities of rib and another site was found to have rib metastasis, but the abnormality on the other site was a benign lesion. Therefore, a total of 3 patients had pure rib metastasis. In addition, 20 patients had bone metastasis at other sites, but their rib abnormalities were not metastatic diseases. The remaining 56 patients were confirmed to have both rib and other site metastasis.

### Risk factors associated with rib metastasis in patients with rib abnormalities

Since rib abnormalities on bone scans were very common with a high false-positive rate, we further analyzed the risk factors for rib metastasis in patients with rib abnormalities.

The rates of rib metastasis among the 324 patients with rib abnormalities stratified by different clinical variables were presented in Table 2. In accordance with the above data on overall bone abnormalities, rib abnormalities in patients with a tumor size > 5 cm were more likely to be rib metastases than those in patients with smaller tumors (*P* = 0.001). Rib abnormalities in patients with lymph node involvement had a trend to be rib metastases compared to those in patients without lymph node involvement, although the difference did not reach statistical significance (*P* = 0.072). Furthermore, concurrent lesions of rib and other sites were significantly associated with a higher risk of rib metastasis than pure rib lesions (*P* < 0.001), and patients with three or more rib lesions were more likely to have bone metastasis than those with one or two rib lesions (*P* < 0.001). The location of rib lesions also had a significant effect on the risk of the metastasis (*P* < 0.001). Similarly, bilateral rib lesions had a higher rate of rib metastases than ipsilateral and contralateral rib lesions (*P* < 0.001). No significant differences were observed between the rates of rib metastasis and other clinical variables, including age, menopausal status, Her2 status, hormone receptors status and molecular subtype (data not shown).

We then performed multivariate logistic regression analyses on the 324 patients with rib abnormalities (Supplement Table 2). Patients with a tumor size > 5 cm (OR = 16.52, 95% CI, 2.03–134.49, *P* = 0.009), ≥3 rib lesions (OR = 2.79, 95% CI, 1.23–6.31, *P* = 0.014), and multiple lesions (OR = 5.9, 95% CI, 1.18–29.57, *P* = 0.031) had significantly higher risk of having rib metastasis compared to their respective reference group. Because the number of rib metastasis in patients with pure rib abnormalities was extremely low (1.2%) and there was only 2 events of rib metastasis in this group, while the rate was 37.7% in 151 patients with concurrent abnormalities, we could not perform this analysis for this variable.

Additionally, multivariate logistic regression analysis was performed on the 151 patients with concurrent abnormalities (Table 3). The abnormalities on bone scans in patients with a tumor size > 5 cm were more likely to be metastatic lesions in comparison to those in patients with a tumor size < 2 cm (OR = 12.32, 95% CI, 0.90–168.90, *P* = 0.060). Moreover, multiple lesions (≥3) were more likely to be metastatic lesions than solitary and double lesions (OR = 2.62, 95% CI, 0.99–6.95, *P* = 0.054). Contralateral rib lesions also had a higher rate of rib metastasis than ipsilateral rib lesions (OR = 5.36, 95%, 0.92–31.15, *P* = 0.061).

### Single or double rib lesions on bone scans

Most rib metastases occur in patients with multiple rib lesions, and single or double rib lesions had a very low risk of rib metastases. Therefore, we performed further detailed analysis of the relationship between clinical variables and the risk of rib metastasis among patients with single or double rib lesions.

**Figure 1:**
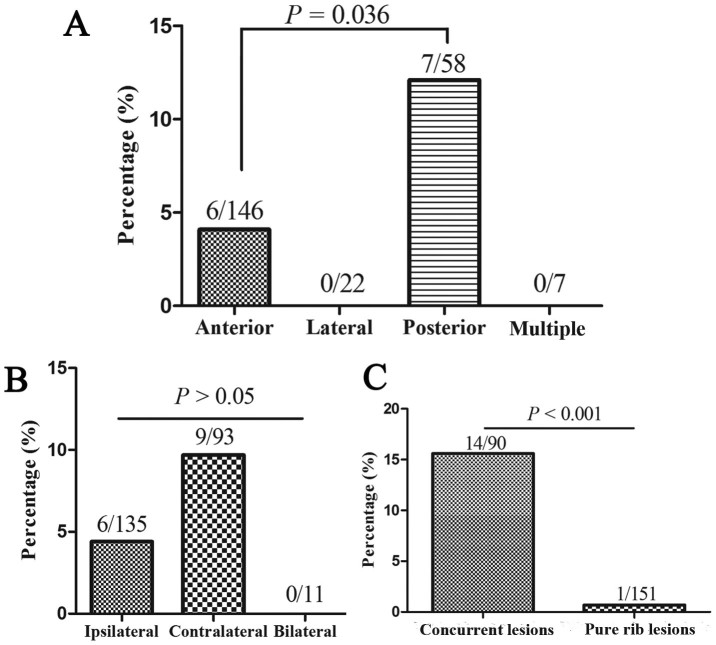
Analysis of rib metastasis in patients with single or double rib lesions on bone scans. (A) posterior rib lesions were more likely to be metastatic than anterior rib lesions; (B) The metastases rates of rib lesions located on ipsilateral, contralateral, and bilateral sides were not significant different; (C) Concurrent lesions of rib and other site on bone scans were more likely to be rib metastatic than pure rib lesions.

A total of 241 patients had single or double lesions on their bone scans. Single or double lesions located on posterior ribs were more likely to be rib metastases than those located on anterior rib (*P* = 0.036, Figure 1). The metastasis rates of rib lesions located on ipsilateral, contralateral, and bilateral sides were not significantly different (*P* > 0.05, Figure 1). The rate of rib metastasis was extremely low in patients with pure single or double rib lesions on their bone scans (0.7%; 95% CI: 0.01%–3.6%). Concurrent lesions of rib and other site on bone scans had a significant higher rate of metastatic disease than pure rib lesions (*P* < 0.001, Figure 1).

## Discussion

Our results showed that in breast cancer patients with bone scan abnormalities, significantly increased rates of bone metastasis were observed in patients with multiple lesions, large tumor size, and lymph node involvement. Patients with concurrent lesions of rib and other sites had a significantly higher rate of metastatic disease than those with only other site lesions. In patients with rib abnormalities, the rate of metastasis was lower in those with pure rib lesions compared to concurrent lesions of rib and other sites. In addition, the rates of rib metastasis were higher in patients with multiple rib lesions, lymph node involvement, and lesions located on bilateral ribs.

^99m^Tc MDP, widely used in SS, is metabolized by osteoclasts^18^. The accumulation of ^99m^Tc MDP on the bone represents hot spot on images, indicating high rate of bone metabolism. Bone metastasis results from destruction caused by the activity of osteoclasts, which can be stimulated by some mediators secreted by tumor^19^. Although bone metastasis is presented as osteolytic, osteoblastic or mixed, both types can be detected on SS with high sensitivity^20^. However, increased rates of metabolism also occur in several other conditions, such as inflammation, fracture and benign bone diseases^21^. To avoid false-positive findings, it is recommended to supplement SS with XR, CT or MRI to confirm the abnormalities on bone scans. Several studies^22^,^23^,^24^,^25^,^26^ have investigated the comparison between SS and ^18^FDG PET/CT on the diagnosis of bone metastasis and suggested that SS is advantageous over PET/CT considering sensitivity, specificity and the cost^27^. Both CT and MRI can contribute to high accuracy of the diagnosis of bone metastasis^28^,^29^. They were used to confirm the osseous destruction of corresponding abnormal sites on bone scans. XR is now rarely used for bone metastasis screening due to its low sensitivity^30^, but it can be helpful when a fracture is suspected.

Patients with multiple lesions on bone scans are associated with a higher rate of metastasis compared to those with single or double lesions. Lesions located on rib combined with other sites are more likely to be bone metastasis than other site lesions. It could be interpreted that increased number of lesions results in higher incidence of metastasis. Previous studies have shown that larger tumor size and positive lymph node were risk factors for developing bone metastasis in breast cancer patients after diagnosis of the disease^1^,^31^,^32^. Consistently, we found that lesions on bone scans of patients with large tumor sizes (>5 cm) and lymph node involvement were more likely to be metastatic.

Our results showed that the rate of metastasis is extremely low (1.2%; 95% CI: 0.1%–4.1%) in patients with pure rib lesions on bone scans. Meanwhile, concurrent lesions of rib and other site were more likely to be rib metastasis compared to the pure rib lesions. Several studies^10^,^11^,^33^ have reported that lesions detected on spine, skull, sternum and pelvis are more likely to be bone metastases than those located on ribs. In addition, a study^34^ has reported that metastasis to rib occurred more frequently in multiple bone metastases than solitary bone metastasis. This extremely low incidence of metastasis in patients with pure rib lesions on bone scans suggest that these patients may only need to be followed up clinically. However, if the rib lesions are detected concurrently with other site lesions, especially those located on spine and sternum, they are very likely to be metastatic disease. Additional radiological examinations should be performed to these patients.

Direct stimulation of surgery and complication of radiotherapy often cause trauma on anterior ribs^35^,^36^, which results in a high rate of false-positive lesions detected on anterior ribs. Moreover, reduced rate of bone metastasis were found in a radiotherapy field owing to radiotherapy^37^,^38^. Therefore, the rates of metastasis were different in different localizations of the rib in patients with rib abnormalities. Multiple rib lesions had the highest rate of rib metastasis, and lesions located on posterior ribs were more likely to be rib metastases than those located on anterior rib. In addition, bilateral rib lesions were associated with a higher rate of metastasis compared to ipsilateral and contralateral rib lesions. The high rate of metastasis in patients with multiple and bilateral rib lesions may be due to increased number of lesions on bone scans.

This study is the largest to date to investigate the relationship between clinical variables and bone metastasis among breast cancer patients with abnormal bone scans. All the patients were identified from an integrated database. Furthermore, the diagnosis of bone metastasis was based on osseous destruction detected on CT or MRI, which made the calculated rate of bone metastasis more accurate. Our results may have important clinical implications: patients with pure rib lesions on bone scans only need follow-up, but a CT or MRI is needed if the rib lesions are detected concurrently with other site lesions.

There are a few limitations in our study. First, as a retrospective study, whether the patients were viscera metastatic is not clear. Prospective study should be conducted for further investigation. Second, biopsy of bone tissue, seldomly used in clinical practice, is not applied to diagnose bone metastasis. Third, other variables, such as clinical manifestation and serum tumor markers, were not available in the present study. Finally, the influence of adjuvant therapies was not investigated in this study.

Our study suggested that pure rib lesions on bone scans were rarely metastatic, but concurrent lesions of rib and other sites were more likely to be metastatic. Therefore, patients with pure rib abnormalities can be followed up, while additional radiological examinations should be performed for patients with concurrent rib lesions and other site lesions on bone scans.

## Methods

### Ethics statement

The present study was approved by the ethics committees of the First Affiliated Hospital with Nanjing Medical University and was in compliance with the Helsinki Declaration. All patients provided written informed consent for their clinical information to be reviewed by us. And all methods were carried out in accordance with the approved guidelines.

### Patients

**Figure 2:**
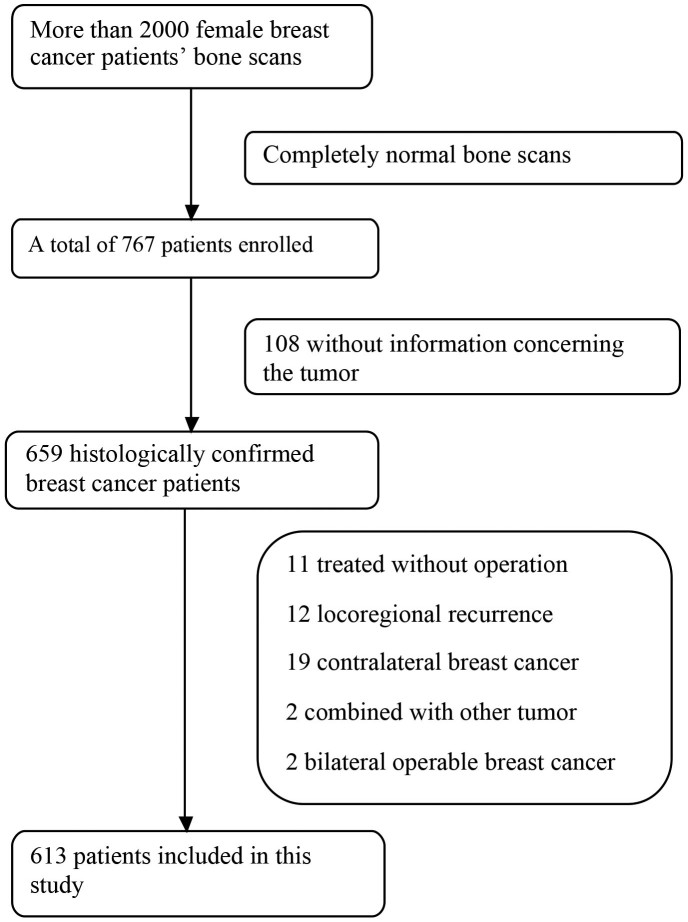
The flow diagram of screened and excluded patients.

To select eligible patients (Figure 2), the database in our hospital was searched for patients enrolled between April 2007 and August 2013. More than 2000 female patients who underwent whole-body SS were found. After excluding patients whose bone scans were completely normal, 767 subjects with bone scan abnormalities were identified for detailed screening. Of these 767 subjects, 108 were excluded due to lack of information about the primary tumor. Patients who matched one of the following criteria were also excluded from our study: (1) breast cancer combined with a synchronous malignant tumor of other site; (2) not surgically treated; (3) locoregional recurrence or contralateral breast cancer; and (4) bilateral operable breast cancer. Finally, 613 female breast cancer patients with bone scan abnormalities were included in this study (Figure 2).

### Skeletal scintigraphy

The SS was performed 2–3 hours after intravenous administration of 740 MBq of ^99m^Tc methylene diphosphonate, using large field of view gamma cameras, equipped with low energy, high resolution collimators. The whole-body images and anterior and posterior views were obtained, as well as selected spot views of suspicious areas. All the images were reviewed by two nuclear medicine physicians independently. Any disagreement would be discussed until a consensus is reached. If the lesion was on rib, the abnormality was further classified according to anatomical location. Being on the same side with the surgery was defined as an ipsilateral lesion. Otherwise, it was defined as a contralateral lesion. If the lesions were located on both sides, they were defined as bilateral lesions. Additionally, the location on the rib could be classified to anterior, lateral and posterior. If the lesions located on at least two locations of the rib, they were defined as multiple.

The data of other radiologic examinations, including XR, CT, MRI and PET, were also collected to confirm the etiology of the increased tracer uptake when it was difficult to diagnose bone metastasis based solely on the results of SS.

### Definitions of bone metastasis

**Figure 3:**
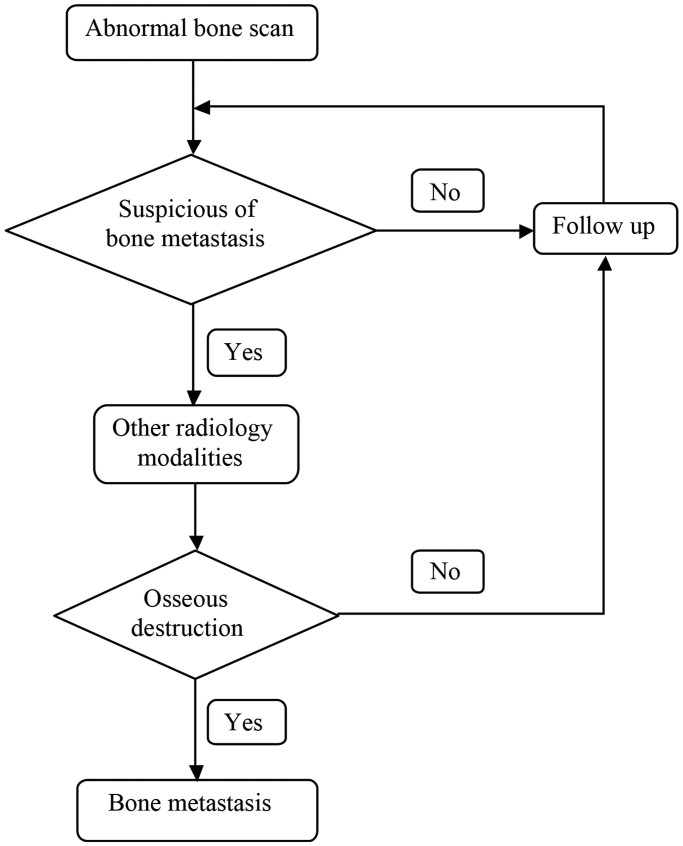
The definition of bone metastasis.

According to the NCCN clinical practice guidelines in oncology for breast cancer, international guidelines for management of metastatic breast cancer from the European School of Oncology (ESO)-MBC Task Force^39^, as well as previous studies^8^,^10^,^13^,^15^,^17^, bone metastasis is confirmed by CT or MRI. Therefore, if the hot spot on bone scan was considered a benign process by the physicians, no additional examinations were needed. The patient would be followed up. On the other hand, if the hot spot on bone scan was diagnosed as a suspicious metastatic disease, radiological result (CT, MRI, PET) at the corresponding site was referred to. The etiology of the abnormality was defined as bone metastasis when osseous destruction was confirmed by radiological modalities. Otherwise the patient would remain on follow-up (Figure 3). During the follow-up, ECT was performed every year or whenever clinical manifestation emerged before the abnormality on SS was confirmed.

### Data collection

Additional data of the 613 patients were collected as follows: (1) demographic data (age and menopausal status); (2) clinical information, such as tumor size, lymph node involvement, hormone receptor status, Her-2 status and molecular subtype; (3) characteristics of the bone scans, including lesion type (rib, other site and both), number of lesions on scans, anatomical location (anterior, lateral, posterior and multiple; ipsilateral, contralateral and bilateral).

### Statistical analysis

Percentiles, median and range were calculated for continuous variables. Univariate analysis was carried out using chi-square test or Fisher's exact test, and logistic regression was used for multivariate analysis. Variables with a P-value < 0.1 in the univariate analysis were further included in multivariate analyses. All P values were two-tailed and P < 0.05 was considered statistical significant. All statistical analyses were performed using STATA version 11.0 (Computer Resource Center, America).

## Supplementary Material

Supplementary Information Supplementary Information

## Figures and Tables

**Table 1:** Analysis of bone metastasis in patients with abnormal bone scans

Variables		Metastatic (%) (n = 126)	Non-metastatic (n = 487)	Total (n = 613)	P-value^1^	OR^2^	95% CI^2^	P-value^2^
**Age**	≤50	62 (21.8)	222	284	0.467			
	>50	64 (19.5)	265	329				
**Menopausal status**	Pre-menopause	50 (20.9)	189	239	0.511			
	Post-menopause	60 (18.7)	261	321				
	NA	16	37	53				
**Tumor size**	≤2 cm	28 (10.9)	229	257	<0.001	Reference		
	2–5 cm	42 (18.4)	186	228		1.53	0.85–2.75	0.158
	>5 cm	9 (39.1)	14	23		3.44	1.10–10.83	0.034
	NA	47	58	105				
**Lymph node**	Negative	34 (12.7)	233	267	0.004	Reference		
	Positive	63 (22.0)	223	286		1.87	1.04–3.34	0.035
	NA	29	31	60				
**Hormone receptor**	Negative	20 (13.6)	127	147	0.517			
	Positive	74 (18.8)	320	394				
	NA	32	40	72				
**Her-2**	Negative	65 (16.6)	327	392	0.723			
	Over-expressing	17 (15.2)	95	112				
	NA	44	65	109				
**Molecular subtype**	Luminal A	33 (16.9)	162	195	0.691			
	Luminal B	29 (17.9)	133	162				
	Her-2	7 (11.9)	52	59				
	Triple negative	12 (14.3)	72	84				
	NA	45	68	113				
**Number of lesions**	1	23 (7.5)	285	308	<0.001	Reference		
	2	22 (16.4)	112	134		0.92	0.37–2.27	0.859
	≥3	81 (47.4)	90	171		3.08	1.32–7.21	0.009
**Lesion type**	Other site lesion	47 (16.3)	242	289	<0.001	Reference		
	Pure rib	2 (1.2)	171	173		/	/	/
	Rib + other site	77 (51.0)	74	151		2.65	1.28–5.51	0.009

NA, not available; OR, odds ratio; CI, confidence interval; 1, calculated by univariate analysis; 2, calculated by multivariate analysis.

**Table 2:** Analysis of rib metastasis in 324 patients with rib lesions on bone scans

Variables		Rib metastasis^1^ (%) (n = 59)	No rib-metastasis^2^ (n = 265)	Total (n = 324)	P-value^3^
**Tumor size**	≤2 cm	8 (6.1)	123	131	0.001
	2–5 cm	21 (17.5)	99	120	
	>5 cm	4 (44.4)	5	9	
	NA	26	38	64	
**Lymph node**	Negative	14 (11.5)	108	122	0.072
	Positive	33 (19.3)	138	171	
	NA	12	19	31	
**Number of rib lesions**	1–2	15 (6.2)	226	241	<0.001
	≥3	44 (53.0)	39	83	
**Concurrent lesions**	No	2 (1.2)	171	173	<0.001
	Yes	57 (37.7)	94	151	
**Localization**	Anterior	11 (6.4)	161	172	<0.001
	Lateral	0 (-)	23	23	
	Posterior	8 (13.1)	53	61	
	Multiple	37(64.9)	20	57	
	NA	3	8	11	
**Relationship with the operative site**	Ipsilateral	10 (6.4)	146	156	<0.001
	Contralateral	11 (11.1)	88	99	
	Bilateral	37 (56.9)	28	65	
	NA	1	3	4	

NA, not available;

^1^The 59 cases included 3 cases with pure rib metastasis and 56 cases with rib + other site metastasis.

^2^The 265 cases included 20 cases with other site metastasis but not rib metastasis and 245 cases without bone metastasis.

^3^Calculated by univariate analysis.

**Table 3:** Multivariate analysis of rib metastasis in 151 patients with concurrent lesions on bone scans

Variables		Rib metastasis (n = 57)	No rib metastasis (n = 94)	Total (n = 151)	P-value^1^	OR^1^	95% CI^1^
**Tumor size**	≤2 cm	8	41	49		Reference	
	2–5 cm	21	34	55	0.140	2.46	0.74–8.16
	>5 cm	4	2	6	0.060	12.32	0.90–168.90
	NA	24	17	41			
**Lymph node**	Negative	12	39	51		Reference	
	Positive	33	47	80	0.996	1.00	0.30–3.38
	NA	12	8	20			
**Number of rib lesions**	1–2	14	76	90		Reference	
	≥3	43	18	61	0.054	2.62	0.99–6.95
**Localization**	Anterior	10	48	58		Reference	
	Lateral	0	11	11	/	/	/
	Posterior	7	17	24	0.654	1.50	0.25–8.81
	Multiple	37	13	50	0.167	4.55	0.53–38.96
	NA	3	5	8			
**Relationship with the operative site**	Ipsilateral	9	51	60		Reference	
	Contralateral	10	26	36	0.061	5.36	0.92–31.15
	Bilateral	37	15	52	0.401	2.51	0.29–21.56
	NA	1	2	3			

NA, not available; OR, odds ratio; CI, confidence interval; 1, calculated by multivariate analysis.
