# Supplementary material for: Risk of metastasis among rib abnormalities on bone scans in breast cancer patients
Source: Sci Rep. 2015 May 5;5:9587. doi: 10.1038/srep09587 (PMC5386210; doi:10.1038/srep09587)
Supplement: Supplementary Information [file srep09587-s1.doc]

**Risk of metastasis among rib abnormalities on bone scans in breast cancer patients**

Qin Li^1※^, Zhiqiang Chen^1※^, Yansheng Zhao^1※^, Xiuqing Li^1^, Hong Pan^1^, Tiansong Xia^1^, Lin Chen^1^, Zhaoqiang Xu^2^, Wenbin Zhou^1^*, Xiaoan Liu^1^*

1 Department of Breast Surgery, The First Affiliated Hospital with Nanjing Medical University, 300 Guangzhou Road, 210029 Nanjing, China

2 Department of Nuclear Medicine, The First Affiliated Hospital with Nanjing Medical University, 300 Guangzhou Road, 210029 Nanjing, China

^※^ These authors contributed equally to this work.

Correspondence and requests for materials should be addressed to: X.L ([liuxiaoan@126.com](mailto:liuxiaoan@126.com)) or W.Z ([zhouwenbin@njmu.edu.cn](mailto:zhouwenbin@njmu.edu.cn)).

**Supplement table 1. Characteristics of the type of bone metastasis in 324 patients with rib lesions on bone scans**

| **Variables** |  | **Pure rib metastatic**  **(n=3)** | **Other sites metastatic**  **(n=20)** | **Rib+other sites metastatic**  **(n=56)** | **Non-metastatic**  **(n=245)** |
| --- | --- | --- | --- | --- | --- |
| **Tumor size** | ≤ 2 cm | 1 | 6 | 7 | 117 |
|  | 2-5 cm | 0 | 6 | 21 | 93 |
|  | >5 cm | 0 | 2 | 4 | 3 |
|  | NA | 2 | 6 | 24 | 32 |
| **Lymph node** | Negative | 3 | 4 | 11 | 104 |
|  | Positive | 0 | 12 | 33 | 126 |
|  | NA | 0 | 4 | 12 | 15 |
| **Number of rib lesions** | 1 | 2 | 10 | 11 | 167 |
|  | 2 | 0 | 2 | 3 | 47 |
|  | ≥3 | 1 | 8 | 43 | 31 |
| **Concurrent lesions** | No | 2 | 0 | 0 | 171 |
|  | Yes | 1 | 20 | 56 | 74 |
| **Lesion location** | Anterior | 2 | 6 | 9 | 155 |
|  | Lateral | 0 | 0 | 0 | 23 |
|  | Posterior | 1 | 5 | 7 | 48 |
|  | Multiple | 0 | 8 | 37 | 12 |
|  | NA | 0 | 1 | 3 | 7 |
| **Relationship with the operative site** | Ipsilateral | 1 | 7 | 9 | 139 |
|  | Contralateral | 2 | 4 | 9 | 84 |
|  | Bilateral | 0 | 9 | 37 | 19 |
|  | NA | 0 | 0 | 1 | 3 |

NA, not available

**Supplement table 2. Multivariate analysis of rib metastasis in 324 patients with rib lesions on bone scans^1^**

| **Variables** |  | **Rib metastasis (%)**  **(n=59)** | **No rib metastasis**  **(n=265)** | **Total**  **(n=324)** | **P-value^2^** | **OR^2^** | **95%CI^2^** |
| --- | --- | --- | --- | --- | --- | --- | --- |
| **Tumor size** | ≤2cm | 8 (6.1) | 123 | 131 |  | Reference |  |
|  | 2-5cm | 21 (17.5) | 99 | 120 | 0.181 | 2.05 | 0.71 - 5.87 |
|  | >5cm | 4 (44.4) | 5 | 9 | 0.009 | 16.52 | 2.03 - 134.49 |
|  | NA | 26 | 38 | 64 |  |  |  |
| **Lymph node** | Negative | 14 (11.5) | 108 | 122 |  | Reference |  |
|  | Positive | 33 (19.3) | 138 | 171 | 0.480 | 1.46 | 0.51 - 4.18 |
|  | NA | 12 | 19 | 31 |  |  |  |
| **Number of rib lesions** | 1-2 | 15 (6.2) | 226 | 241 |  | Reference |  |
|  | ≥3 | 44 (53.0) | 39 | 83 | 0.014 | 2.79 | 1.23 - 6.31 |
| **Lesion location** | Anterior | 11 (6.4) | 161 | 172 |  | Reference |  |
|  | Lateral | 0 (-) | 23 | 23 | / | / | / |
|  | Posterior | 8 (13.1) | 53 | 61 | 0.549 | 1.62 | 0.33 - 7.81 |
|  | Multiple | 37(64.9) | 20 | 57 | 0.031 | 5.90 | 1.18 - 29.57 |
|  | NA | 3 | 8 | 11 |  |  |  |
| **Relationship with the operative site** | Ipsilateral | 10 (6.4) | 146 | 156 |  | Reference |  |
|  | Contralateral | 11 (11.1) | 88 | 99 | 0.084 | 3.80 | 0.84 - 17.33 |
|  | Bilateral | 37 (56.9) | 28 | 65 | 0.168 | 3.34 | 0.60 - 18.63 |
|  | NA | 1 | 3 | 4 |  |  |  |

1, The variable “concurrent lesions” were not analyzed;

2, calculated by multivariate analysis;

NA, not available; OR, odds ratio; CI, confidence interval;
